# miR-548aj-3p and miR-3127-3p suppress RANKL-facilitated inflammatory cytokines and catabolic factor in osteoarthritis and rheumatoid arthritis

**DOI:** 10.7150/ijms.110812

**Published:** 2025-07-28

**Authors:** Yu-Han Wang, Chin-Horng Su, Li-Chai Chen, Ju-Fang Liu, Chun-Hao Tsai, Yi-Chin Fong, Chih-Yuan Ko, Hsien-Te Chen, Lun-Chien Lo, Chih-Hsin Tang

**Affiliations:** 1 Department of Pharmacology, School of Medicine, China Medical University, Taichung, Taiwan.; 2 Institute of Medicine, Chung Shan Medical University, Taichung, Taiwan.; 3 Department of Orthopedics, Asia University Hospital, Taichung, Taiwan.; 4 Department of Beauty Science, National Taichung University of Science and Technology, Taichung, Taiwan.; 5 Department of Pharmacy, Tajen University, Pingtung, Taiwan.; 6 School of Oral Hygiene, College of Oral Medicine, Taipei Medical University, Taipei, Taiwan.; 7 Department of Sports Medicine, College of Health Care, China Medical University, Taichung, Taiwan.; 8 Department of Orthopedic Surgery, China Medical University Hospital, Taichung, Taiwan.; 9 Department of Orthopedic Surgery, China Medical University Beigang Hospital, Yunlin, Taiwan.; 10 Graduate Institute of Biomedical Sciences, China Medical University, Taichung, Taiwan.; 11 School of Chinese Medicine, College of Chinese Medicine, China Medical University, Taichung, Taiwan.; 12 Department of Chinese Medicine, China Medical University Hospital, Taichung, Taiwan.; 13 Department of Medical Laboratory Science and Biotechnology, Asia University, Taichung, Taiwan.; 14 Chinese Medicine Research Center, China Medical University, Taichung, Taiwan.

**Keywords:** osteoarthritis, rheumatoid arthritis, miRNA, inflammatory cytokine, matrix metalloproteinase

## Abstract

Osteoarthritis (OA) and rheumatoid arthritis (RA) are highly prevalent joint diseases globally. The common pathological features include synovial inflammation, swelling, joint destruction, and bone remodeling. Arthritis development is associated with joint inflammation, particularly in inflamed synovial cells. Synovial inflammation contributes to joint destruction. The receptor activator of nuclear factor kappa-B ligand (RANKL) is a vital factor that is linked to the activity of osteoclasts and the erosion of bone. Increased levels of RANKL play a role in the course of arthritis. Adverse effects and individual differences in therapeutic efficacy are limits of arthritis medications. More effective treatment and drug options are needed to improve disease progression. miRNAs directly modulate gene transcription as a potential option for arthritis therapeutics. The GEO dataset from the synovium of normal, OA, and RA patients indicated that the expression levels of RANKL were upregulated and related to arthritis features. We found that RANKL stimulation in OA and RA synovial fibroblasts decreased miR-548aj-3p and miR-3127-3p expression and enhanced interleukin-1 beta (IL-1β), interleukin-6 (IL-6), and matrix metalloproteinase-13 (MMP-13) production by using quantitative reverse transcription polymerase chain reaction (RT-qPCR) and enzyme-linked immunosorbent assay (ELISA). miRNA sequencing analysis and target prediction tools identified that miR-548aj-3p and miR-3127-3p regulate IL-1β, IL-6, and MMP-13 expression and are inhibited by RANKL stimulation. Administration of miR-548aj-3p and miR-3127-3p mimics significantly inhibited RANKL-induced expression of IL-1β, IL-6, and MMP-13 at both the mRNA and protein levels. We propose a potentially efficacious miRNA therapeutic approach for the treatment of arthritis, with a specific focus on OA and RA.

## 1. Introduction

Osteoarthritis (OA) and rheumatoid arthritis (RA) are prevalent joint diseases globally. The number of OA cases worldwide has more than doubled from 1990 to 2019, reaching 527.81 million people^1^. According to statistics as of 2020, the global number of people living with RA has increased by 1.2 times since 1990, reaching 17.6 million individuals 2. OA is a degenerative joint disorder characterized by cartilage degradation and bone remodeling, driven by chondrocyte activity in the articular cartilage and low-grade inflammatory responses from surrounding synovial cells 3. RA is an autoimmune disease characterized by synovitis and a systemic immune response that results in the degradation of cartilage and bone 4. The elevated levels of rheumatoid factor (RF) and anti-cyclic citrullinated peptide (anti-CCP) serve as diagnostic indicators for RA patients 5. Inflammatory cytokines, such as interleukin-1 beta (IL-1β) and interleukin-6 (IL-6), were elevated in OA joints but remained lower than in RA joints 6, 7. Additionally, the inflamed synovium also secretes matrix metalloproteinases (MMPs), which are enzymes responsible for breaking down the extracellular matrix, particularly articular cartilage 8, 9. Matrix metalloproteinase-13 (MMP-13) is the key MMP to cleavage type II collagenase in OA and RA 10, 11. While the processes of onset for OA and RA might differ, there are certain similarities in the mechanisms, such as inflammation and cartilage degradation. It not only affects the mobility of patients but also exacerbates the progression of the disease. Hence, discovering therapeutic medications that efficiently suppress the synthesis of inflammatory factors and MMPs in the inflamed synovium would be advantageous for OA and RA treatment.

The receptor activator of nuclear factor κB ligand (RANKL) is a crucial factor linked to osteoclast activity and bone erosion in arthritis diseases, including OA and RA 12. Aberrant mechanical loading on the OA joint contributes to the loss of cartilage proteoglycans and the resorption of subchondral bone, potentially due to elevated local expression of RANKL 13. Significantly high RANKL expression in OA cartilage may serve as an important chemoattractant for osteoclasts and may participate in the degradation of bone and cartilage collagen during the progression of OA 14. Patients with OA demonstrate markedly increased RANKL expression in their synovial tissue relative to healthy individuals 15. Furthermore, RANKL levels in RA synovial cells are three times higher than those in OA synovial cells 16. This increased expression plays a crucial role in initiating joint inflammation. RANKL interacts with pro-inflammatory cytokines, such as tumor necrosis factor-alpha (TNF-α) and IL-6, to create an inflammatory milieu that affects the synovium and other joint structures 17. Previous studies indicated that the production of RANKL by chondrocytes may play a role in the degradation of cartilage in RA 18. Activated immune cells stimulate synovial fibroblasts (SF cells) to produce pro-inflammatory cytokines and tissue-degenerative proteins, resulting in the inflammation and joint destruction observed in RA 19. In both OA and RA, the raised levels of RANKL in the synovium and cartilage can potentially stimulate joint inflammation and lead to cartilage degradation 20, 21. Therefore, it is crucial to thoroughly investigate the impact of RANKL on the inflamed synovium.

microRNAs (miRNAs) are a class of small non-coding RNA molecules that play important regulatory roles in gene expression by binding to target mRNAs. In recent years, the field of miRNA biology has rapidly expanded. The widespread deregulation of miRNAs has been observed in various diseases, including arthritis 22, 23. For instance, miR-150-5p not only binds to vascular endothelial growth factor (VEGF) to promote angiogenesis but also interacts with vascular cell adhesion protein 1 (VCAM-1) to attract immune cells, specifically monocytes, infiltrating into synovial cells and amplifying the inflammatory response in OA 24-26. IL-1β and transforming growth factor-β (TGF-β) suppress the production of miR-140 in OA chondrocytes, resulting in the breakdown of the articular cartilage matrix by upregulating the expression of catabolic factors such as integrin and metalloproteinase with thrombospondin motifs-5 (ADAMTS5), MMP-13, insulin-like growth factor-binding protein-5 (IGFBP5), and RAS like proto-oncogene A (RALA) 27. miR-146a suppresses the proliferation of fibroblast-like synoviocytes and reduces inflammatory response by downregulating the toll-like receptor 4 (TLR4)/nuclear factor kappa-B (NF-κB) pathway in RA 28. miR-23a inhibits interleukin-17 (IL-17)-induced NF-κB activation by reducing the expression of pro-inflammatory mediators such as IL-6, monocyte chemoattractant protein-1 (MCP-1), and matrix metalloproteinase-3 (MMP-3) in chondrocytes from RA patients, thereby alleviating cartilage matrix degradation and joint structure destruction 29. Hence, identifying miRNAs that can simultaneously prevent articular cartilage degradation and control the inflammatory response in both OA and RA would be extremely beneficial for the treatment of arthritis. Bioinformatics analysis revealed that RANKL expression was significantly higher in the synovium of OA and RA patients compared to healthy individuals 30, 31. We found that treatment of OA synovial fibroblasts (OASF cells) and RA synovial fibroblasts (RASF cells) with RANKL significantly enhanced the production of proinflammatory cytokines (IL-1β and IL-6) and increased the secretion of catabolic factor MMP-13, while TNF-α and matrix metalloproteinase-1 (MMP-1) did not significantly increase in protein levels. Our miRNA sequencing (miRNA-seq) data revealed that miR-548aj-3p and miR-3127-3p are the most downregulated miRNAs in OASF cells and RASF cells following RANKL stimulation. However, the role of miR-548aj-3p and miR-3127-3p regarding OA and RA is still unknown. To realize the role of miR-548aj-3p and miR-3127-3p in OA and RA, we predicted miR-548aj-3p and miR-3127-3p binding sites target the 3' untranslated regions (3'UTRs) of IL-1β, IL-6, and MMP-13. Transfected miR-548aj-3p and miR-3127-3p mimics in OASF and RASF cells significantly blockade RANKL-induced IL-1β, IL-6, and MMP-13 expression. These results suggest that a combination of miR-548aj-3p and miR-3127-3p mimics therapeutic medicines to inhibit the inflammatory response in arthritis and prevent the degradation of articular cartilage. We propose a combination therapy of miR-548aj-3p and miR-3127-3p mimics as a new approach for the treatment of arthritis.

## 2. Materials and Methods

### 2.1. Cell culture

OA synovial tissue specimens were obtained from patients who underwent knee replacement for Kellgren-Lawrence (KL) grade 4 OA between June 2023 and June 2024 (n = 5). The age of OA patients ranged from 43 to 63 years old, with a male-to-female ratio of 2:3. All patients provided informed consent granted by the Institutional Review Board (IRB) of China Medical University Hospital. OASF cells were digested with type II collagenase and then cultured as previously described 32. Human fibroblast-like synoviocytes (normal SF cells) were purchased from Cell Applications, Inc. (CA, USA). Human RA synovial fibroblast cell line MH7A (RASF cells) was isolated from an RA patient (53 years old, female) and purchased from Riken (Ibaraki, Japan).

Normal SF cells and OASF cells were cultured in Dulbecco's modified Eagle's medium (DMEM). (Gibco, NY, USA) RASF cells were cultured in RPMI-1640 medium (Gibco, NY, USA). All of the cell culture media were supplemented with 10 % fetal bovine serum (FBS) (ATLAS Biologicals, CO, USA) and penicillin/streptomycin at 100 U/mL. All cells were used between passages 3 and 5 to ensure the accuracy and reproducibility of the results from the experiment 33.

### 2.2. Bioinformatic analysis

The GSE89408 dataset from the Gene Expression Omnibus (GEO) database was used to examine the expression of RANKL and arthritis-related factors in the healthy donor (n=28), OA (n=22), and RA (n=152) patient samples of synovial tissue.

Ingenuity pathway analysis (IPA) was conducted on differentially expressed genes (DEGs) from GSE89408 dataset to identify biological pathways significantly impacting mRNA expression in arthritis. DEGs were identified by using a cutoff threshold of log2-transformed fold change (log2FC) ≥ |1| and a p-value < 0.05.

The OASF cells and RASF cells were divided into a control group (Ctrl) and a RANKL treatment group (RANKL) dependently. The Bioconductor suite DESeq2 (V1.26.0) was used to analyze miRNA-seq data, identifying differentially expressed genes based on log2FC ≥ |1.5| and p-value < 0.05 34. GOSeq performs functional enrichment analysis, providing insights into the GO functional enrichment of differentially expressed microRNA target genes in miRNA-seq data 35.

### 2.3. Real-time quantitative polymerase chain reaction amplification

The human recombinant RANKL was purchased from Prospec (Rehovot, Israel, cyt-334).OASF cells and RASF cells were treated with RANKL for 24 h, and total RNA was extracted using TRIzol reagent. Reverse transcription was carried out using 2 μg of total RNA, which was transcribed into complementary DNA (cDNA) using the M-MLV RT kit (Thermo Fisher, Taipei, Taiwan) for mRNA and the Mir-X™ miRNA First-Strand Synthesis kit (Terra Bella Avenue; Mountain View, CA, USA) for miRNA 25. Quantitative real-time polymerase chain reaction (RT-qPCR) was performed using the Taqman® One-Step QRT-PCR Master Mix (Applied Biosystems) and analyzed using the StepOnePlus™ Real-Time PCR (RT-qPCR) System (Applied Biosystems) 36.

### 2.4. miRNA filtering and transfection

We used the miRDB, miRWalk, and TargetScan databases to predict miRNA and mRNA 3'UTR binding regions, focusing on the seed region of a microRNA, which is a 7-8 nucleotide sequence within these binding regions 37.

The miR-548aj-3p and miR-3127-3p mimics, along with their respective control, the negative control (NC) mimic (miRNA negative control with no homology to any known miRNA or mRNA sequences), were acquired from AllBio Science (Taipei, Taiwan). The diverse dosages (5, 10, and 25 nM) of miR-548aj-3p mimic, miR-3127-3p mimic, and their respective control NC mimic were transfected into OASF cells and RASF cells using Lipofectamine 2000, respectively 25. After 24 h transfection, OASF and RASF cells were treated with 50 ng/ml RANKL for another 24 h, and then the RNA expression levels were examined by the RT-qPCR system 38, 39.

### 2.5. ELISA assay

All of the ELISA kits (DY201-05, DY206-05, DY210-05, DY901B-05, and DY511) include IL-1β, IL-6, TNF-α, MMP-1, and MMP-13 were acquired from R&D Systems (M.N., USA). The conditioned medium (CM) from normal SF, OASF, and RASF cells was collected after 24 hours of incubation with or without RANKL (50 ng/ml). The experimental methods were performed according to the manufacturer's instructions, and the samples (n=3 for each group) were incubated overnight at room temperature without dilution 40.

### 2.6. Statistical analysis

The results are presented as the mean ± standard deviation (S.D.). Statistical analysis was performed using t-tests and one-way ANOVA to compare the differences between the experimental groups. A *p*-value < 0.05 was considered statistically significant.

## 3. Results

### 3.1. Increased RANKL levels correlate with molecules regulating proinflammatory cytokines and catabolic factors in synovial tissues from OA and RA patients

**Figure 1:**
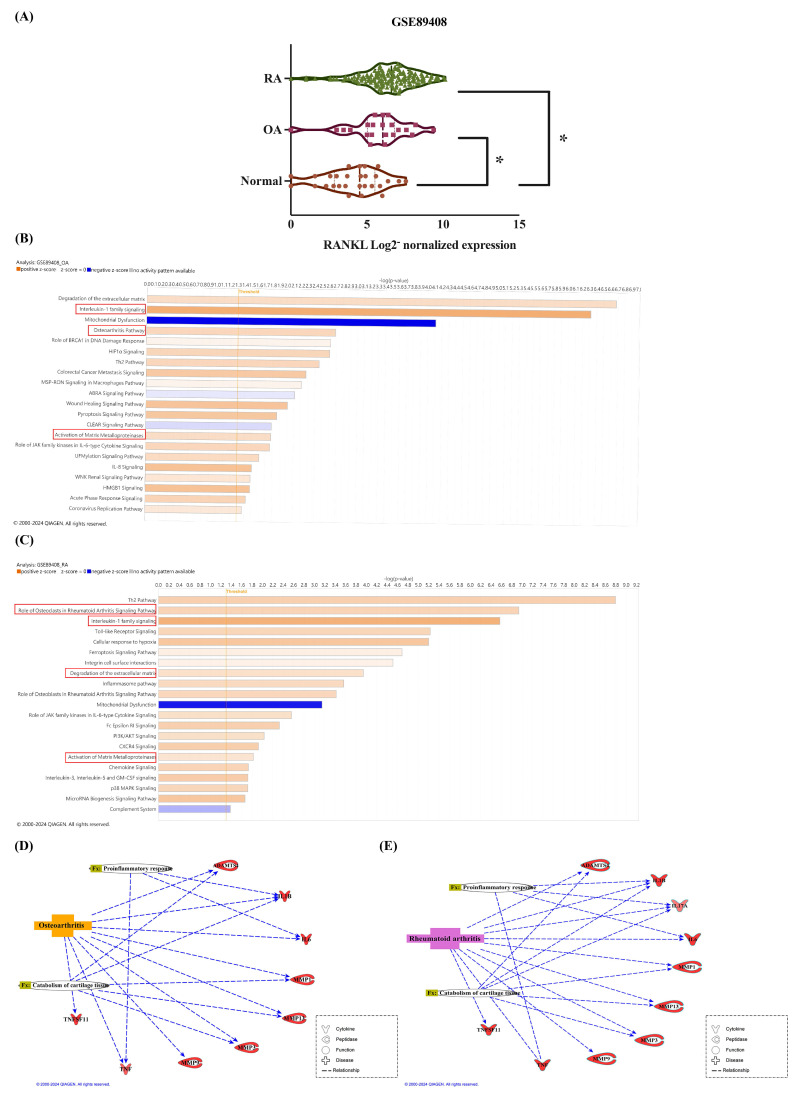
** Higher levels of RANKL are related to the pro-inflammatory response and cartilage tissue catabolism in synovial tissues from OA and RA patients.** (A) The expression of RANKL in synovial tissue expression profiles was analyzed from the GEO database, including healthy tissues (n=28), OA tissues (n=22), and RA tissues (n=152). (B-C) The IPA analysis identified enriched biological pathways in the GSE89408 database. Colors represent the extent of z-scores, indicating pathway activity based on changes in gene expression. A z-score > 2 (orange) suggests significant activation, while a z-score < -2 (blue) indicates significant inhibition. (D-E) The interaction network between differentially expressed genes, biological functions, and diseases was analyzed from the GSE89408 database. (The dotted line means direct connections.) Results are expressed as the means ± SD. *p < 0.05 vs. normal SF cells.

Inflammatory cytokines stimulate the production of RANKL, resulting in the degradation of articular cartilage and bone erosion in arthritis. The impact of increased RANKL levels in SF cells on the progression of arthritis remains unclear. Our analysis retrieved from the GEO database revealed elevated RANKL expression in the synovium of OA and RA patients compared to healthy donors (Figure 1A). To uncover mechanisms underlying increased RANKL expression in OA and RA, we used the IPA software to analyze the canonical pathways involved in the GSE89408 database. The z-score predicts the activation or inhibition of pathways, regulators, or functions based on gene expression from data. The results showed that interleukin-1 family signaling, the osteoarthritis pathway, and the matrix metalloproteinase pathway were predicted to be activated in OA patients (Figure 1B). Besides, the role of osteoclasts in the rheumatoid arthritis signaling pathway, interleukin-1 family signaling, degradation of extracellular matrix, and activation of matrix metalloproteinases are predicted to activate in RA individuals (Figure 1C). The genes analyzed from the GSE89408 database include IL-1β, IL-6, and TNF-α, which are associated with proinflammatory responses in OA and RA, while L-17 is exclusively related to RA (Figure 1D). MMPs (MMP-1, MMP-3, MMP-9, and MMP-13) and extracellular proteases (ADAMTS-4 and ADAMTS-5) are the genes associated with catabolism of cartilage tissue in OA and RA (Figure 1E).

**Figure 2:**
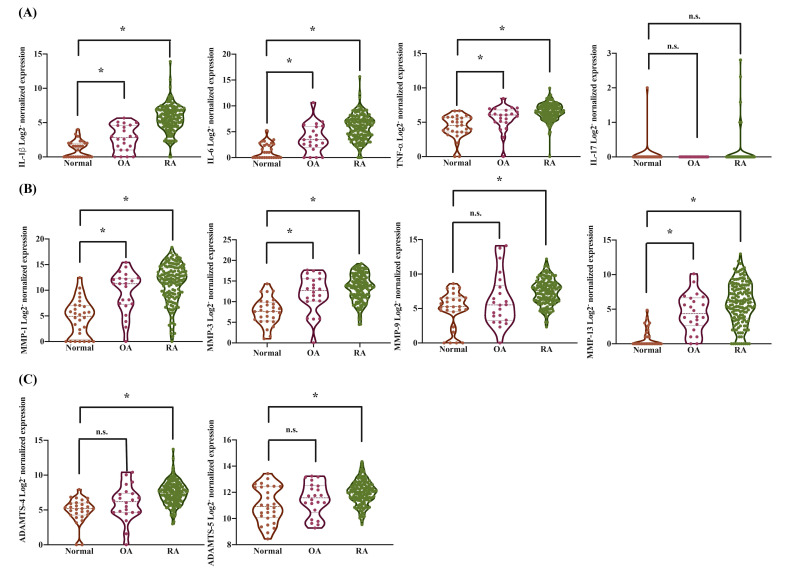
** The expression levels of pro-inflammatory cytokines and cartilage degradation enzymes in synovial tissues from the GEO database.** Gene expression levels in synovial tissues from normal individuals, OA, and RA patients were analyzed from the GSE89408 database. The gene expression data were standardized using base 2 logarithms. (A) The expression levels of pro-inflammatory cytokines, including IL-1β, IL-6, TNF-α, and IL-17, were analyzed. (B) The expression levels of MMPs, including MMP-1, MMP-3, MMP-9, and MMP-13, were evaluated. (C) The expression levels of proteases, including ADAMTS-4 and ADAMTS-5, were examined. Results are expressed as the means ± SD. *p < 0.05 vs. normal SF cells; n.s., not significant.

We further observed that pro-inflammatory cytokines, including IL-1β (2.37-fold increase in OA, 20.15-fold in RA), IL-6 (3.17-fold in OA, 19.5-fold in RA), and TNF-α (1.29-fold in OA, 1.5-fold in RA), were significantly elevated in synovial tissues from OA and RA patients compared to those from healthy donors (Supplementary Figure S1). This analysis was conducted using synovial tissue samples from 28 healthy individuals, 22 OA patients, and 152 RA patients. However, there was no significant upregulation in the expression of IL-17 observed in the analysis (Figure 2A). MMP-1, MMP-3, MMP-9, and MMP-13 play critical roles in the pathogenesis of both OA and RA and contribute to joint degradation 41. These MMPs are key mediators of disease progression and potential therapeutic targets. Therefore, we observed a significant upregulation of cartilage degradation-related enzymes, specifically MMP-1 (16.17-fold increase in OA, 39.31-fold in RA), MMP-3 (20.36-fold increase in OA, 25.9-fold in RA), and MMP-13 (9.31-fold increase in OA, 16.51-fold in RA), in synovial tissues from OA and RA patients compared to those from healthy donors (Figure 2B-C and Supplementary Figure S2). In contrast, the expression of MMP-9 (1.33-fold increase in OA, 2.86-fold in RA), ADAMTS-4 (1.65-fold increase in OA, 2.96-fold in RA), and ADAMTS-5 (1.07-fold increase in OA, 1.14-fold in RA) was increased in RA but did not show a significant change in OA (Figure 2B-C and Supplementary Figure S2). These results revealed that increased RANKL levels in the synovium of arthritis patients are associated with molecules that regulate arthritic features, including proinflammatory cytokines, catabolic factors, and processes leading to bone and cartilage destruction.

### 3.2. RANKL stimulation enhances IL-1β, IL-6, and MMP13 production in OA and RA

**Figure 3:**
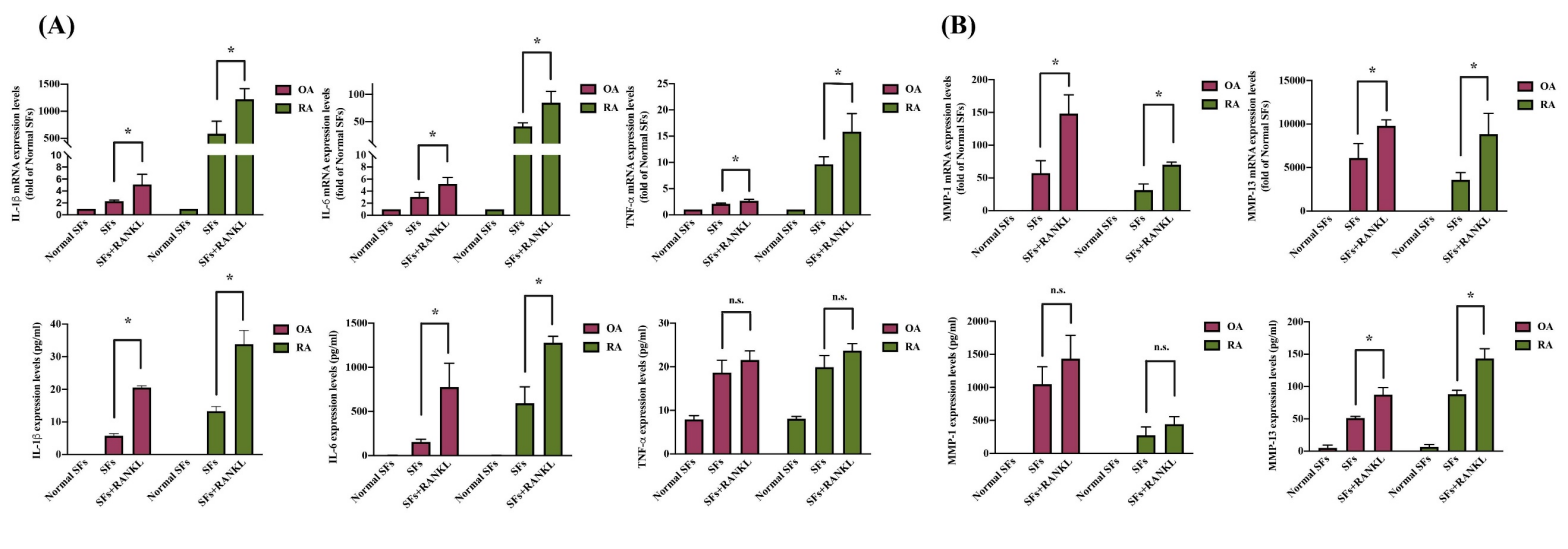
** RANKL enhances IL-1β, IL-6, and MMP-13 in OA and RA synovial cells.** Synovial cells isolated from OA and RA patients were treated with RANKL (50 ng/ml) for 24 h, and then RNA and cell supernatant were collected for RT-qPCR and ELISA assays. (A) Total RNA was isolated from normal SF (n=3), OASF (n=3), and RASF cells (n=3). The expression levels of IL-1β, IL-6, and MMP-13 were examined by RT-qPCR. (B) The CM from normal SF (n=3), OASF (n=3), and RASF cells (n=3) were collected, and then the protein levels were measured at the absorbance of 450nm by using ELISA kits. Sample protein concentration was calculated using the standard curve formula based on their OD values. Results are expressed as the means ± SD. *p < 0.05 vs. OASF cells or RASF cells.

Inflammation response and cartilage degradation are pivotal in the pathogenesis of arthritis, particularly in synovial cells 42, 43. We observed that pro-inflammatory cytokines IL-1β, IL-6, and TNF-α levels and the cartilage degradation enzymes MMP-1 and MMP-13 are substantially increased in SF cells derived from OA and RA patients compared to normal SF cells (Figure 3A-B). RANKL stimulation significantly increased both mRNA and protein expression of IL-1β, IL-6, and MMP-13 in OASF and RASF cells (Figure 3A-B). However, TNF-α and MMP-1 mRNA and protein levels had no significant difference in response to RANKL treatment compared to both OASF and RASF cells (Figure 3A-B). We next investigated how RANKL specifically modulates the expression of IL-1β, IL-6, and MMP-13 but not TNF-α and other MMPs. Our findings indicate that RANKL stimulation especially enhances the expression of pro-inflammatory cytokines (IL-1β and IL-6) and the cartilage-degrading enzyme MMP-13 in both OASF and RASF cells.

### 3.3. miR-548aj-3p and miR-3127-3p were significantly downregulated in OASF and RASF cells treated with RANKL

**Figure 4:**
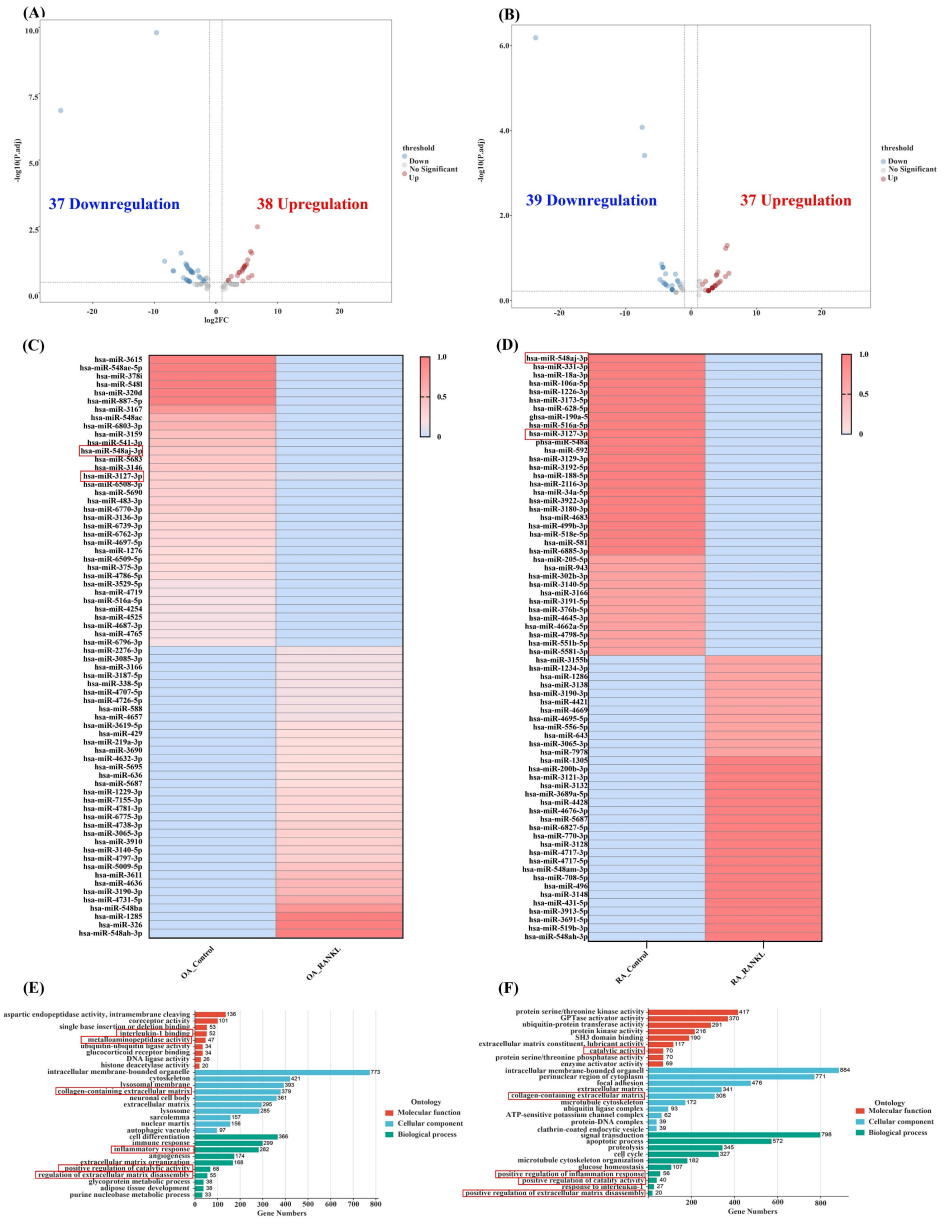
**Transcriptomic analysis from miRNA-seq.** (A-B) Volcano plots illustrate the differentially expressed miRNAs in OASF or RASF cells stimulated with RANKL compared to unstimulated OASF and RASF cells. miRNAs with an absolute log2 fold change greater than 1.5 are highlighted, with upregulated miRNAs shown in red and downregulated miRNAs in blue. (C-D) The heatmap shows the expression of miRNAs in control and RANKL groups within OA and RA miRNA-seq. The color gradient represents expression levels, with red indicating higher expression and blue indicating lower expression. (E-F) GO classification analysis of the target genes of differentially expressed miRNAs between the RANKL-treated and control groups in OASF and RASF cells. The target genes were categorized into molecular function (red), cellular component (blue), and biological process (green) classifications.

miRNA inhibits mRNA transcription by binding to the 3' UTR of target mRNA and participates in the regulation of biological pathways. To investigate the effects and regulatory pathways of RANKL in OASF and RASF cells, we performed miRNA-seq analysis. In OA, we identified 75 differentially expressed miRNAs after RANKL stimulation in OASF cells, which included 38 upregulated and 37 downregulated miRNAs compared to unstimulated OASF cells. (Figure 4A). Among them, miR-548aj-3p expression significantly decreased by more than 4-fold after RANKL stimulation in OASF cells, while miR-3127-3p expression significantly decreased by 1.6-fold. Moreover, we observed 76 differentially expressed miRNAs in RANKL-stimulated RASF cells compared to unstimulated RASF cells, with 37 miRNAs upregulated and 39 miRNAs downregulated (Figure 4B). In particular, miR-548aj-3p expression was significantly lowered by 23.8-fold, and miR-3127-3p expression was significantly reduced by 3.8-fold after RANKL stimulation in RASF cells. The heatmap displays the differentially expressed miRNAs from our miRNA-seq analysis results (Figure 4C-D). The GO analysis results showed that the molecular function categories of differentially expressed miRNA targets in OA miRNA-seq include interleukin-1 binding and metalloaminopeptidase activity, whereas the targets identified in RA miRNA-seq were associated with catalytic activities. The cellular component categories involve collagen-containing extracellular matrix in both OA and RA. Besides, biological processes are involved in inflammation response, extracellular matrix disassembly, and catalytic activity regulation in OA and RA (Figure 4E-F).

**Figure 5:**
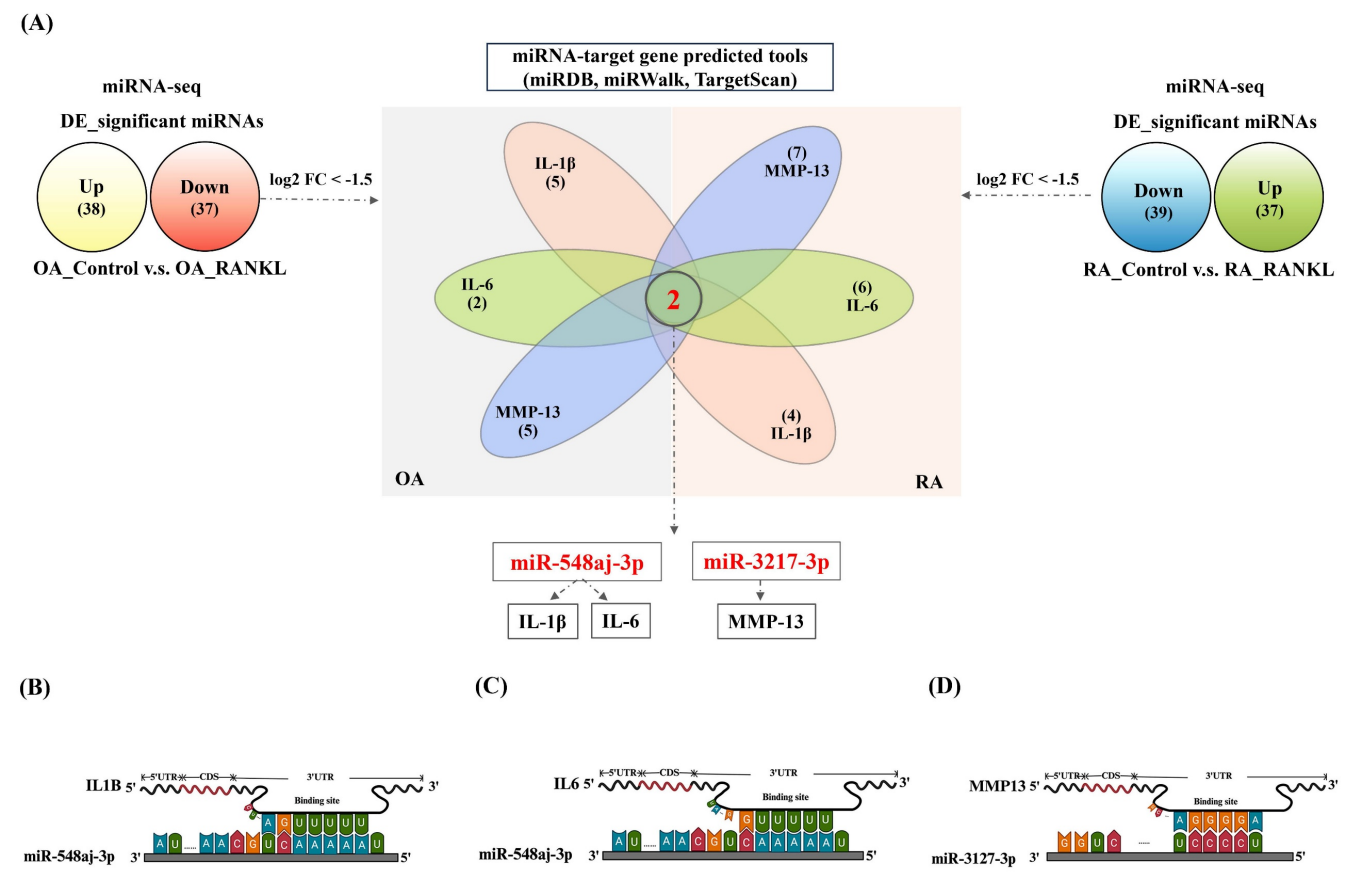
** miR-548aj-3p and miR-3127-3p were downregulated after RANKL stimulation and predicted to regulate inflammatory cytokines and cartilage-degrading enzymes.** (A) The diagram depicts the differentially expressed miRNAs regulated by RANKL stimulation. The Venn diagram highlights miRNAs predicted to interact with IL-1β, IL-6, and MMP-13 using publicly available miRNA-target gene databases (miRDB, miRWalk, and TargetScan). (B-C) Schematic representation of the predicted binding sites of miR-548aj-3p on the 3' untranslated regions (3'UTRs) of IL-1β and IL-6. (D) The predicted binding site of miR-3127-3p on the 3'UTR of MMP-13.

We further filtered the miRNAs that were significantly downregulated under the predefined threshold of log2FC ≤ -1.5 after RANKL treatment from the OA and RA miRNA-seq. Additionally, we used miRNA target prediction tools, including miRDB, miRWalk, and TargetScan, to filter these downregulated miRNAs for predicted interactions with IL-1β, IL-6, or MMP-13. The results revealed that miR-548aj-3p was the most significant downregulated miRNA in OASF cells and RASF cells, and it interacts with the proinflammatory cytokines IL-1β and IL-6. Furthermore, miR-3127-3p was also substantially downregulated in OASF and RASF cells and was predicted to interact with the matrix metalloproteinase MMP-13 (Figure 5A). The illustration showed the target sites for miR-548aj-3p and miR-3127-3p, predicted by the open-source platform TargetScan. (Figure 5B-D). These results suggest that miR-548aj-3p and miR-3127-3p may act as regulators of RANKL-induced IL-1β, IL-6, and MMP-13 expression in OASF and RASF cells.

### 3.4. Upregulation of miR-548aj-3p and miR-3127-3p impedes RANKL-induced IL-1β, IL-6 and MMP-13 production in OA and RA

**Figure 6:**
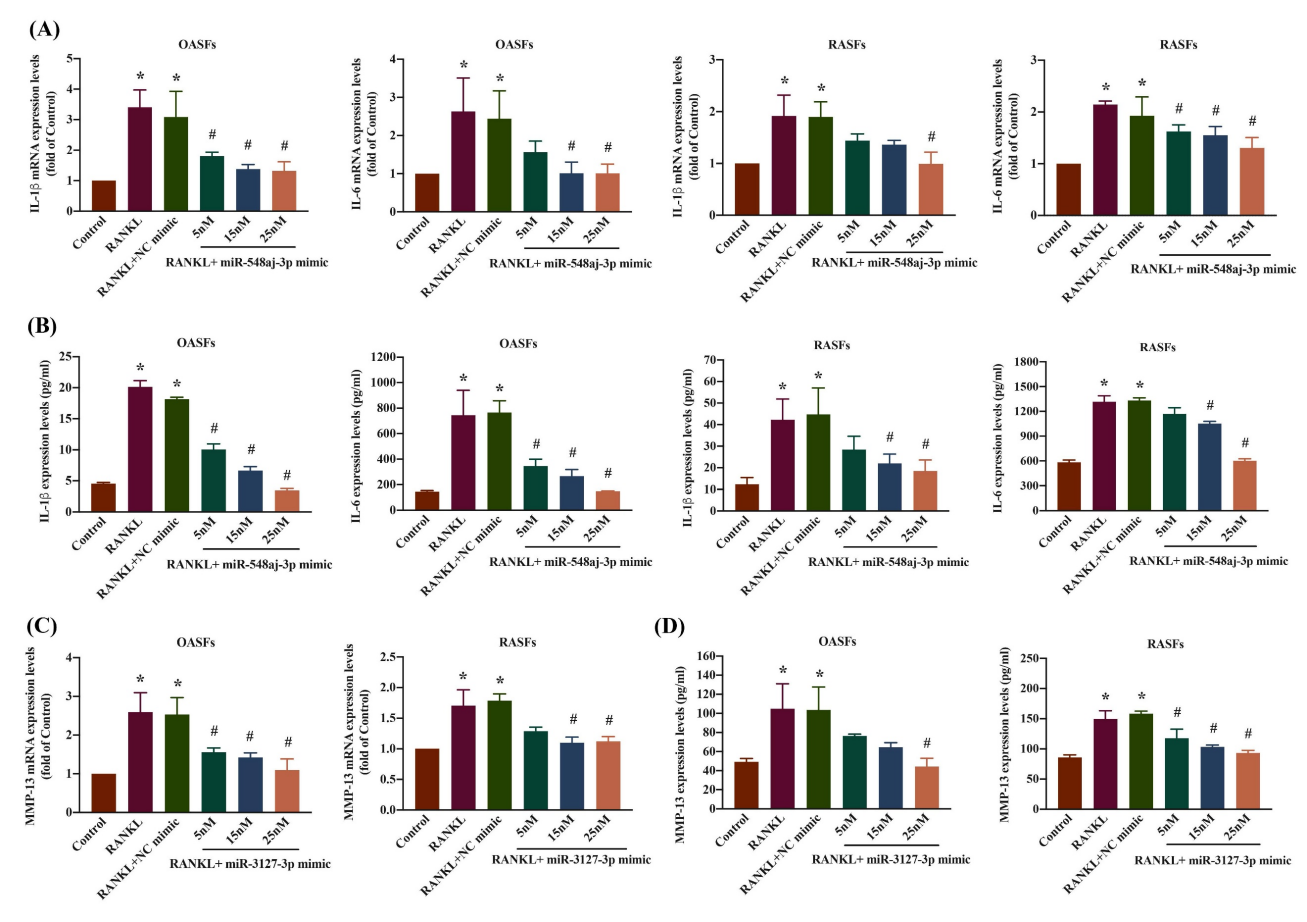
** miR-548aj-3p and miR-3127-3p regulated pro-inflammatory cytokines and matrix metalloproteinase production underlying RANKL stimulation.** (A-B) OASF (n=3) and RASF cells (n=3) were transfected with diverse dosages of miR-548aj-3p mimic (5-25 nM) for 24 h and then treated with RANKL for another 24 h. Total RNAs were extracted, and IL-1β and IL-6 mRNA were detected by using RT-qPCR. Cell supernatant was collected, and then IL-1β and IL-6 protein levels were measured by ELISA assay. (C-D) Fibroblasts derived from the synovial tissue of individuals diagnosed with OA (n=3) and RA (n=3) were transfected with miR-3127-3p mimic at concentrations ranging from 5 to 25 nM for 24 h, followed by treatment with RANKL for an additional 24 h. The mRNA expression of MMP-13 was assessed using RT-qPCR, and the protein levels of MMP-13 were measured by ELISA. Results are expressed as the means ± SD. *p < 0.05 vs. control group. # *p* < 0.05 vs. RANKL group.

We evaluated the involvement of miR-548aj-3p and miR-3127-3p in the signaling pathways regulating RANKL-induced upregulation of inflammatory cytokines (IL-1β and IL-6) and the cartilage-degrading enzyme MMP-13 in OASF and RASF cells. We transfected OASF and RASF cells with miR-548aj-3p mimics, miR-3127-3p mimics, or NC mimics and observed significant, dose-dependent effects on IL-1β, IL-6, and MMP-13 mRNA and protein expression using RT-qPCR and ELISA assays. The results revealed that administration of miR-548aj-3p mimic significantly inhibited IL-1β and IL-6 mRNA and protein levels (Figure 6A-B), whereas miR-3127-3p mimics greatly suppressed MMP-13 mRNA and protein synthesis (Figure 6C-D) in both OASF and RASF cells. The findings suggest that the increased expression of miR-548aj-3p and miR-3127-3p inhibits the production of IL-1β, IL-6, and MMP-13 induced by RANKL.

## Discussion

Arthritis (OA and RA) is a high-prevalence joint disease 44, 45. The onset of OA typically arises from various factors, including prolonged weight-bearing activities, joint trauma, and advanced age. RA is a systemic inflammatory disease characterized by a strong inflammatory response that attacks joints and other tissues. Despite the pathophysiology of OA and RA being different, both diseases exhibit pathological features such as synovial inflammation, cartilage degeneration, bone resorption, and remodeling. These pathological features have similar regulatory pathways at the molecular level affecting arthritis progression. IL-1β, TNF-α, and IL-6 are the central pro-inflammatory cytokines that participate in arthritis 46, 47. They are produced abundantly within inflamed synovial cells and possess potent pro-inflammatory properties that enable them to stimulate chondrocytes and osteoblasts to secrete large amounts of other inflammatory mediators, triggering the characteristic inflammatory symptoms of arthritis, including joint pain, swelling, and redness 48, 49. In arthritis, pro-inflammatory cytokines (IL-1β, IL-6, and TNF-α) initiate and prolong the production of more degradative enzymes such as MMPs 50, 51.

In our analysis of publicly available transcriptomic datasets from the GEO database, we observed significantly higher fold changes in the expression of pro-inflammatory cytokines such as IL-1β and IL-6, as well as MMPs, in synovial tissue samples from RA patients compared to those from OA patients (Supplementary Figure S1). These datasets included a substantial number of biologically distinct clinical samples (28 from healthy controls, 22 from OA patients, and 152 from RA patients). The substantially elevated expression patterns of inflammatory cytokines and MMPs observed in RA compared to OA reflect the distinct pathophysiological mechanisms underlying these two joint disorders 52-55. RA is a systemic autoimmune disorder characterized by persistent synovial inflammation and aberrant activation of immune pathways 56. This chronic inflammatory state drives the sustained overproduction of pro-inflammatory cytokines, which upregulate MMPs, leading to progressive joint destruction 57. In contrast, osteoarthritis is primarily a degenerative joint disease resulting from mechanical stress and age-related cartilage deterioration 58. It typically involves low-grade inflammation, with only modest elevations in cytokine and MMP expression 59. Moreover, RANKL secreted from inflamed SF cells, in combination with TNF-α and IL-6 from activated immune cells, promotes the differentiation of macrophages and preosteoclasts into osteoclasts that are specifically adapted for the breakdown of bone tissue 49, 60. Evidence indicates that pro-inflammatory cytokines such as TNF-α, IL-1β, IL-6, and IL-17 regulate RANKL 61. However, few studies discuss how RANKL forms a positive feedback loop with pro-inflammatory factors, further amplifying inflammatory responses in arthritis. Our study revealed that RANKL stimulation significantly enhanced the production of IL-1β, IL-6, and MMP-13 but not TNF-α, ADAMTS-4, or ADAMTS-5 in OASF and RASF cells compared to their unstimulated counterparts (Figure 3A and B). The results suggest that TNF-α, ADAMTS-4, and ADAMTS-5 production may not be directly regulated by RANKL stimulation in OASF and RASF cells. These results suggest that high levels of RANKL in either OASF cells or RASF cells especially impact the secretion of IL-1β, IL-6, and MMP-13.

The pharmaceutical treatments for OA and RA are to alleviate symptoms and halt or slow down the disease activity. Non-steroidal anti-inflammatory drugs (NSAIDs), such as ibuprofen and naproxen, are the first-line therapy in arthritis and demonstrate more potent efficacy than paracetamol and opioids in alleviating pain and enhancing functionality in OA patients 62, but their prolonged usage is associated with obvious safety concerns 63, 64. In RA, NSAIDs result in symptom alleviation but do not have any impact on the disease progression 65. Methotrexate (MTX) is a conventional disease-modifying anti-rheumatic medication (DMARD) along with glucocorticoid utilized for RA treatment 66. Nevertheless, an enormous percentage of patients find that MTX monotherapy is inadequate in effectively controlling the RA symptoms and has adverse side effects such as hepatic fibrosis, pulmonary fibrosis, and renal impairment 67. Advances in comprehending the underlying mechanisms of arthritis have enabled the identification of multiple potential targets for treatment that are involved in inflammation, cartilage repair and breakdown, and bone remodeling 68. Biological DMARDs or disease-modifying OA drugs (DMORDs) are the newer therapeutic strategies for arthritis. The anti-inflammatory biologic agents, such as the IL-1β receptor antagonist (AMG 108) and the IL-1β antagonist (lutikizumab), were the first biologics investigated by randomized controlled trials (RCTs) for OA treatment. However, the trials showed that AMG 108 and lutikizumab did not demonstrate superiority over placebo in reducing symptoms and joint damage in patients 69-71. Moreover, TNF-α inhibitors (infliximab and adalimumab) and IL-6 inhibitor (tocilizumab) have undergone RCTs in OA and RA patients 72-74. In individuals with existing hand OA, infliximab therapy results in a slight statistically significant decrease in the risk of OA progression, particularly in the distal interphalangeal joints 75. In patients with hand OA and inflamed joints, long-term adalimumab treatment (lasting more than 12 months) reduced the incidence of joint erosion but did not improve symptoms 76. An insufficient response to TNF-α inhibitors has been observed in RA, but the underlying reasons remain not fully understood. On the contrary, tocilizumab is a humanized monoclonal antibody targeting IL-6R, commonly used to treat RA, and its treatment can reduce disease activity 65. Our results revealed that RANKL regulates the production of IL-1β and IL-6 by inhibiting miR-548aj-3p in OASF and RASF cells. Besides the anti-inflammatory biologic agents, there have been drugs targeting cartilage, such as the ADAMTS-5 inhibitor (GLPG1972/S201086), which is currently being investigated in a global phase II study involving patients with knee OA 62. The MMP inhibitors (PG-116800) have been reported with reversible musculoskeletal adverse events in OA patients 77. Our study did not observe that RANKL stimulation to SF cells from OA and RA patients induced ADAMTS production. However, we found that RANKL induced the release of the cartilage catabolic factor MMP-13 through suppressing levels of miR-3127-3p. Taken together, these results demonstrated that RANKL stimulates the secretion of pro-inflammatory cytokines and matrix-degrading enzymes in OASF and RASF cells. The synergistic effect of these anti-inflammatory biologic agents with miR-548aj-3p and miR-3127-3p mimics may contribute to improving the inflammatory response in the joint while also halting cartilage degradation and bone destruction.

miRNAs serve as post-transcriptional regulators of gene expression, interacting with the 3'UTR of target mRNAs to suppress their expression 78, 79. Thus, miRNAs participate in various biological processes, including inflammation and metabolism 80, 81. MicroRNA-146a suppresses the inflammatory responses by regulating the TLR4/NF-kB signaling pathway in RA fibroblast-like synoviocytes^28^. miR-27 inhibits hepatic gluconeogenesis by directly targeting transcription factor forkhead box protein-1 (FOXO1) 82. Our study revealed that miR-548aj-3p affects the production of proinflammatory cytokines IL-1β and IL-6, whereas miR-3127-3p regulates the transcription of MMP-13 in response to RANKL stimulation in OASF and RASF cells. Our innovative approach proposes the use of miR-548aj-3p and miR-3127-3p mimics as therapeutics. This strategy holds promise for simultaneously targeting key pathological mechanisms of arthritis (OA and RA), specifically by inhibiting synovial inflammation and preventing cartilage degeneration. However, there are still certain challenges that must be resolved, including the occurrence of severe immune-related adverse effects, the necessity for specific organ targeting, and the requirement for fine-tuned dosing.

Primary normal SF cells are typically derived from synovial tissue obtained during surgery or trauma-related procedures. However, obtaining sufficient quantities of these cells presents significant challenges due to the invasive procedures required for sample collection and associated ethical constraints. In the case of RA, the effective use of DMARDs and biologic agents in early and moderate disease stages has greatly reduced the need for surgical intervention 83-85, thereby limiting access to primary synovial tissue from RA patients. These challenges substantially restrict the ability to perform comprehensive intracellular analyses using primary normal SF cells and RASF cells. Normal SF cells we used in this study, derived from the synovial lining of healthy joints, provide a robust cell model for studying arthritis diseases as a control group 86, 87. An immortalized RASF cell line (MH7A) is widely used as a substitute for primary RASF cells due to its stable phenotype, reproducibility, and cytokine responsiveness 88-90. Using normal SF cells and MH7A cells represents a well-established and accepted approach to studying intracellular signaling and molecular mechanisms in joint diseases 91, 92. These models allow for ethical, reproducible studies of intracellular regulating mechanisms in *in vitro* studies. Thus, we employed commercially available normal SF cells and the MH7A cell line for* in vitro* experiments. In conclusion, despite limitations in the availability of primary cell samples, our integrated approach—including the use of established cell models and validation via large-scale transcriptomic datasets—allowed us to robustly demonstrate that RANKL enhances IL-1β, IL-6, and MMP-13 production in both OASF and RASF cells through the regulation of miR-548aj-3p and miR-3127-3p. Here, we present a potentially effective therapeutic strategy for treating arthritis, specifically targeting OA and RA. The potential therapeutic use of miR-548aj-3p and miR-3127-3 is based on RNA interference (RNAi) strategies, representing a promising avenue for precision medicine approaches that may minimize adverse effects commonly observed with conventional treatments.

## Supplementary Material

Supplementary figures and tables.
