# Supplementary material for: miR-548aj-3p and miR-3127-3p suppress RANKL-facilitated inflammatory cytokines and catabolic factor in osteoarthritis and rheumatoid arthritis
Source: Int J Med Sci. 2025 Jul 28;22(14):3650–63. doi: 10.7150/ijms.110812 (PMC12434817; doi:10.7150/ijms.110812)
Supplement: Supplementary file 1 — Supplementary figures and tables. [file ijmsv22p3650s1.pdf]

miR-548aj-3p and miR-3127-3p suppress RANKL-facilitated inflammatory cytokines and catabolic factor in osteoarthritis and rheumatoid arthritis

Yu-Han Wang, Chin-Horng, Li-Chai Chen, Ju-Fang Liu, Chun-Hao Tsai, Yi-Chin Fong, Chih-Yuan Ko, Hsien-Te Chen, Lun-Chien Lo, Chih-Hsin Tang

This file includes:

Supplementary data

Supplementary Table

Supplementary data

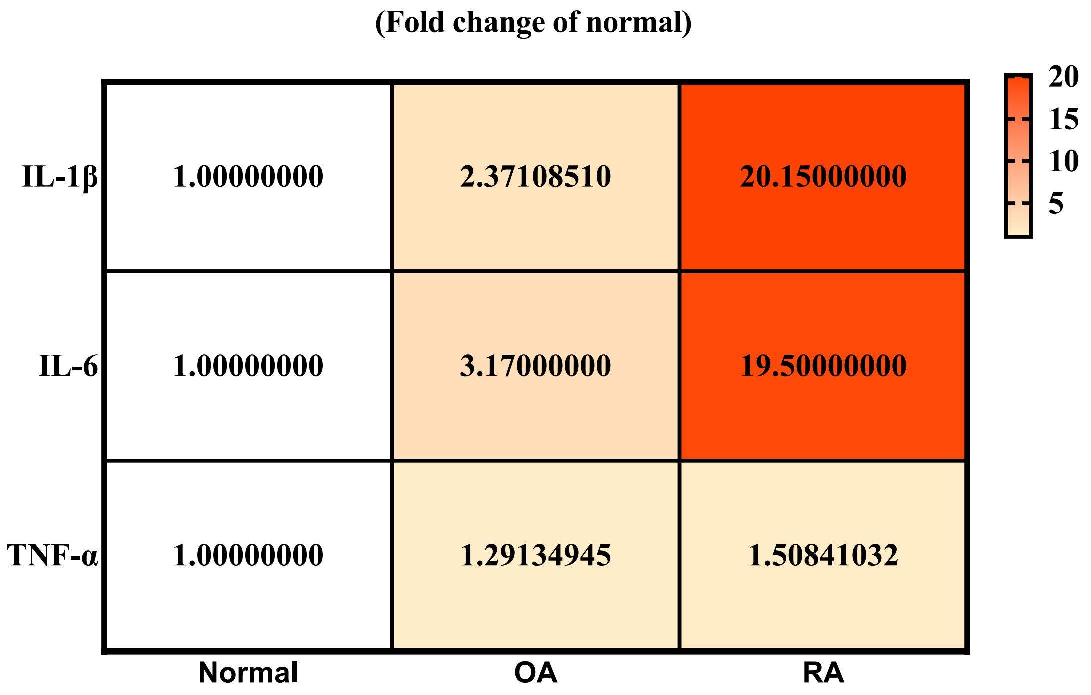

Supplementary Figure S1. The heatmap illustrates the fold changes in expression levels of IL-1β, IL-6, and TNF-α in OA or RA synovial tissues compared to those of healthy individuals[1]. A higher fold change corresponds to a more intense red color.

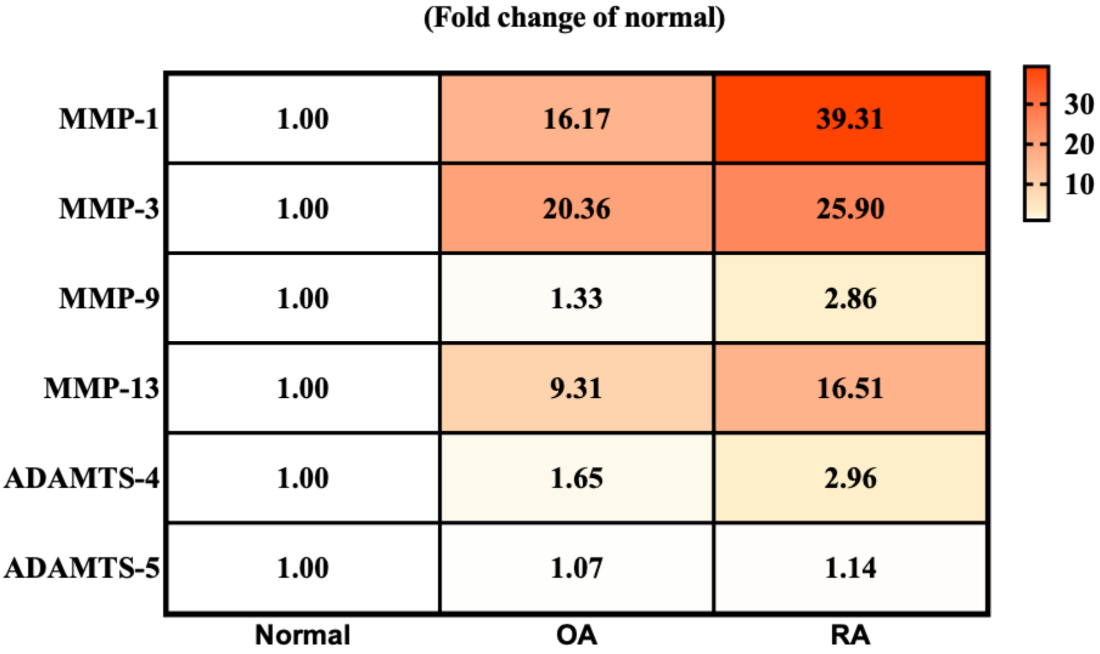

Supplementary Figure S2. The heatmap displays the comparative expression levels of catabolic factors (MMP-1, MMP-3, MMP-9, MMP-13, ADAMTS-4, and ADAMTS-5) in OA and RA synovial tissues versus those in tissues from healthy subjects, with fold changes indicated. [1]. A greater fold change results in a deeper red color.

Supplementary Table S1

The list of abbreviations used in this article.

**Abbreviations**	**Full name**
3’UTRs	3’ untranslated regions
Abbreviations	Full name
ADAMTS5	metalloproteinase with thrombospondin motifs-5
anti-CCP	anti-cyclic citrullinated peptide
cDNA	complementary DNA
CM	conditioned medium
DEGs	differentially expressed genes
DMARD	disease-modifying anti-rheumatic medication
DMEM	Dulbecco’s modified Eagle’s medium
DMORDs	disease-modifying OA drugs
ELISA	enzyme-linked immunosorbent assay
FBS	fetal bovine serum
FOXO1	forkhead box protein-1
GEO	Gene Expression Omnibus
IGFBP5	insulin-like growth factor-binding protein-5
IL-17	interleukin-17
IL-1β	interleukin-1 beta
IL-6	interleukin-6
IPA	Ingenuity pathway analysis
IRB	Institutional Review Board
KL	Kellgren-Lawrence
log2FC	log2-transformed fold change
MCP-1	monocyte chemoattractant protein-1
miRNAs	microRNAs
miRNA-seq	miRNA sequencing
MMP-1	matrix metalloproteinase-1
MMP-13	matrix metalloproteinase-13
MMP-3	matrix metalloproteinase-3
MTX	Methotrexate
NC	negative control
NF-κB	nuclear factor kappa-B
Normal SF cells	human fibroblast-like synoviocytes
NSAIDs	Non-steroidal anti-inflammatory drugs

OA	osteoarthritis
OASF cells	OA synovial fibroblasts
RA	rheumatoid arthritis
RALA	RAS like proto-oncogene A
RANKL	receptor activator of nuclear factor kappa-B ligand
RASF cells	human RA synovial fibroblast cell line MH7A
RCTs	randomized controlled trials
RF	rheumatoid factor
RNAi	RNA interference
RT-qPCR	quantitative reverse transcription polymerase chain reaction
SF cells	synovial fibroblasts
TGF-β	transforming growth factor-β
TLR4	toll-like receptor 4
TNF-α	tumor necrosis factor-alpha
VCAM-1	Vascular cell adhesion protein-1
VEGF	vascular endothelial growth factor

Supplementary Table S2

The list of mRNA primer sequences used in RT-qPCR.

**Gene**	**Forward (5'…………3')**	**Reverse (5'…………3')**
IL-1β	ATGATGGCTTATTACAGTGGCAA	GTCGGAGATTCGTAGCTGGA
IL-6	AGACAGCCACTCACCTCTTCAG	TTCTGCCAGTGCCTCTTTGCTG
TNF-α	CCTCTCTCTAATCAGCCCTCTG	GAGGACCTGGGAGTAGATGAG
MMP-1	AAAATTACACGCCAGATTTGCC	GGTGTGACATTACTCCAGAGTTG
MMP-13	CCAGACTTCACGATGGCATTG	GGCATCTCCTCCATAATTTGGC
GAPDH	ACCACAGTCCATGCCATCAC	TCCACCACCCTGTTGCTGTA

Supplementary Table S3

The list of miRNA mimic sequences used in this study.

**mimic ID**	**Sequence（5'…………3')**
Negative control	UUCUCCGAACGUGUCACGUTT<br>ACGUGACACGUUCGGAGAATT
has-miR-548aj-3p mimics	UAAAAACUGCAAUUACUUUUA<br>AAAGUAAUUGCAGUUUUUAUU
has-miR-3127-3p mimics	UCCCCUUCUGCAGGCCUGCUGG<br>AGCAGGCCUGCAGAAGGGGAUU

Reference

[1] M.I. Love, W. Huber, S. Anders, Moderated estimation of fold change and dispersion for RNA-seq data with DESeq2, Genome Biol 15(12) (2014) 550.
